# Factors associated with cancer survival disparities among Aboriginal and Torres Strait Islander peoples compared with other Australians: A systematic review

**DOI:** 10.3389/fonc.2022.968400

**Published:** 2022-09-15

**Authors:** Paramita Dasgupta, Veronica Martinez Harris, Gail Garvey, Joanne F. Aitken, Peter D. Baade

**Affiliations:** ^1^ Viertel Cancer Research Centre, Cancer Council Queensland, Brisbane, QLD, Australia; ^2^ School of Public Health, Faculty of Medicine, University of Queensland, Brisbane, QLD, Australia; ^3^ School of Public Health and Social Work, Faculty of Health, Queensland University of Technology, Brisbane, QLD, Australia; ^4^ Institute for Resilient Regions, University of Southern Queensland, Brisbane, QLD, Australia; ^5^ Centre for Data Science, Faculty of Science, Queensland University of Technology, Brisbane, QLD, Australia; ^6^ Menzies Health Institute Queensland, Griffith University, Southport, QLD, Australia

**Keywords:** Aboriginal and Torres Strait Islander, inequalities, Australia, cancer, survival

## Abstract

**Background:**

While cancer survival among Aboriginal and Torres Strait Islander peoples has improved over time, they continue to experience poorer cancer survival than other Australians. Key drivers of these disparities are not well understood. This systematic review aimed to summarise existing evidence on Aboriginal and Torres Strait Islander cancer survival disparities and identify influential factors and potential solutions.

**Methods:**

In accordance with PRISMA guidelines, multiple databases were systematically searched for English language peer-reviewed articles on cancer survival by Aboriginal and Torres Strait Islander status published from 1/1/2008 to 4/05/2022. Observational studies presenting adjusted survival measures in relation to potential causal factors for disparities were included. Articles were screened independently by two authors. Included studies were critically assessed using Joanna Briggs Institute tools.

**Results:**

Thirty population-based and predominantly state-level studies were included. A consistent pattern of poorer unadjusted cancer survival for Aboriginal and Torres Strait Islander peoples was evident. Studies varied widely in the covariates adjusted for including a combination of socio-demographics, cancer stage, comorbidities, and treatment. Potential contributions of these factors varied by cancer type. For lung and female breast cancer, adjusting for treatment and comorbidities reduced the survival disparity, which, while still elevated was no longer statistically significant. This pattern was also evident for cervical cancer after adjustment for stage and treatment. However, most studies for all cancers combined, or colorectal cancer, reported that unexplained survival disparities remained after adjusting for various combinations of covariates.

**Conclusions:**

While some of the poorer survival faced by Aboriginal and Torres Strait Islander cancer patients can be explained, substantial disparities likely to be related to Aboriginal determinants, remain. It is imperative that future research consider innovative study designs and strength-based approaches to better understand cancer survival for Aboriginal and Torres Strait Islander peoples and to inform evidence-based action.

## 1 Introduction

Australia has high overall cancer survival rates compared to international benchmarks (1). While survival rates among Aboriginal and Torres Strait Islanders, the First Nations peoples of Australia following a cancer diagnosis have improved over time (2–6), they continue to experience poorer survival than other Australian cancer patients (2, 7, 8). For example, the five-year relative survival during 2012-16 for all cancers combined was 54% for Aboriginal and Torres Strait Islander peoples and 68% for other Australians (7). Aboriginal and Torres Strait Islander peoples also had lower 5-year survival for a range of individual cancer types including female breast, cervical, colorectal, lung, liver and head and neck cancers. They are also more likely to be diagnosed with and die from all cancers combined and some cancer types such as liver, lung, head and neck and colorectal cancers than other Australians (7). These differences likely reflect a range of factors including differences between the two populations in the prevalence of risk and/or protective factors such as smoking, alcohol consumption, access to health-care services, receipt of treatment and uptake of screening and diagnostics testing.

Over the past decade, various initiatives have been implemented to address the Aboriginal and Torres Strait Islander peoples disparity in health, including cancer, such as The National Aboriginal and Torres Strait Islander Cancer Framework, a policy document to guide and direct the many individuals, communities, organisations and governments whose combined efforts are required to address disparities and improve cancer outcomes for Aboriginal and Torres Strait Islander peoples (9); the establishment of regional cancer centres; and improved access to health care through Indigenous-specific primary health care centres (4, 10, 11). However, the persistent and continuing disparity in survival serves to highlight the complexity of reducing this disparity.

Factors contributing to these survival disparities are complex and multifactorial. Aboriginal and Torres Strait Islander peoples experience disadvantage across a range of socioeconomic and health indicators, have lower average life expectancy, and are more likely to live in more remote areas than other Australians (2), with Australian oncology services typically concentrated in major cities (12). There is also a substantial travel burden for cancer patients in more remote areas to attend oncology care in cities (13, 14). There is evidence that Aboriginal and Torres Strait Islander peoples face additional logistical, systemic, health system and social barriers, including perceptions about cancer and its treatment and a lack of culturally appropriate care (2, 15–18) that likely contribute to disparate health outcomes.

Various multi-dimensional models have been developed that provide a framework to depict the wide array of health determinants potentially relevant in explaining the disparities in cancer outcomes between Indigenous (including Aboriginal and Torres Strait Islander peoples) and non-Indigenous peoples (17, 19). A range of factors including structural inequities and institutionalised discrimination translate into differences in determinants of health, exposures, and opportunities (at multiple levels); access to care and quality of care all of which contribute to Indigenous cancer inequities. These factors occur across the cancer continuum from diagnosis to treatment and beyond and may help explain the survival disparities faced by Aboriginal and Torres Strait Islanders peoples following a cancer diagnosis. While various studies have examined specific aspects of these disparities in Australia for a range of cancer types, there is no comprehensive published synthesis of their findings. A previous systematic review was limited to breast cancer (15) while one government report described inequalities in cancer outcomes by Aboriginal and Torres Strait Islander status and socio-economic factors (20). This has limited the ability of relevant stakeholders to identify gaps in knowledge, formulate strategic research, and implement appropriate action.

This systematic review was undertaken to synthesise contemporary evidence on survival disparities by Aboriginal and Torres Strait Islander status and identify the factors that may explain these disparities.

## 2 Methods

### 2.1 Ethics

Ethics approval was not required for this systematic review as the study involved secondary analysis of previously published peer-reviewed articles.

### 2.2 Patient involvement

No patients were directly involved in the development of the research questions, choosing the outcome measures of interest, study design and implementation or interpretation of results.

### 2.3 Review protocol

This systematic review was designed, conducted, and reported in accordance with the Preferred Reporting Items for Systematic Reviews and Meta Analyses (PRISMA) guidelines (**Supplemental checklist**) (21). The review protocol is registered in the International Prospective Register of Systematic Reviews-PROSPERO (www.crd.york.ac.uk/PROSPERO, registration number: CRD42021278842).

### 2.4 Review question

The review question, defined following the structured PECO framework (22), was: in those diagnosed with cancer (Participants), what are the factors associated with the survival outcome disparities (Outcome) faced by Aboriginal and Torres Strait Islander peoples (Exposure) compared with other Australians (Comparator)?

### 2.5 Literature searches

The electronic bibliographic databases: PubMed, Embase, CINAHL, Web of Science and the Australian Indigenous Health*InfoNet* (23) were systematically searched for all indexed articles from 1 January 2008 to focus on more contemporary estimates, that are likely to be more relevant rather than historical estimates and to reduce the impact of temporal improvements in the quality of Aboriginal and Torres Strait identification. Our starting point of 2008 was also consistent with a previous grey literature government report on inequalities in cancer outcomes by socio-economic factors among Aboriginal and Torres Strait Islander peoples (20). Final searches were undertaken on 4 May 2022.

Search strategies used selected subject headings and key words related to “Australia”, and “neoplasms” combined with Aboriginal and Torres Strait Islander search terms of “Indigenous”‘, “Torres Strait Islander” or “Aboriginal” and outcome measures of “survival” or “mortality” (**Supplementary Materials Table S1.1**).

### 2.6 Inclusion and exclusion criteria

Studies were included if they met the following criteria:

1. Setting was Australia; and
2. Population comprised adults (aged ≥15 years) diagnosed with invasive cancer; and
3. Study cohort included Aboriginal and Torres Strait Islander Australians; and
4. Outcome measure was survival after a cancer diagnosis; and
5. Presented quantitative survival estimates by Aboriginal and Torres Strait Islander status; and
6. Assessed factors associated with Aboriginal and Torres Strait Islander peoples survival disparities; and
7. Reported adjusted effect estimates enabling comparison between Aboriginal and Torres Strait Islanders peoples and other Australians from survival regression models.

The scope of the review was limited to English-language peer-reviewed original research articles. Reviews, editorials, commentaries, and conference abstracts were excluded although their reference lists were checked to identify additional potentially relevant articles.

The term study is used throughout this review to refer to an individual published manuscript.

### 2.7 Aboriginal and Torres Strait Islander status

Information on Aboriginal and Torres Strait Islander status in Australian population-based cancer registry data is primarily based on self-reported data at point of care in hospitals and other health services and on information in death records (24, 25). The completeness, consistency, and quality of information on Aboriginal and Torres Strait identification is not consistent between jurisdictions nor over time. In past years, quality of information has been poor, however, in recent years, cancer registries have improved the accuracy and completeness of identification of Aboriginal and Torres Strait Islander peoples across their whole data collection by using data linkage to combine self-reported data from multiple administrative datasets and applying algorithms to derive the most plausible data (24–26).

### 2.8 Study screening

All searched articles from the citation databases were exported into Endnote X7. No software was used for data screening. Two reviewers (VMH, PD) independently screened by hand the titles and abstracts of all unique articles identified by the queries. Discrepancies were discussed and resolved through consensus. The full text of all articles deemed potentially relevant and those whose abstracts and titles provided insufficient information were then retrieved. Two reviewers (VMH, PD) independently selected the final articles for inclusion into the review after a full and comprehensive reading (reasons noted). All decisions were compared and discussed and, if necessary, the senior author (PB) was consulted.

### 2.9 Data extraction

Data from all selected studies were extracted by VMH into a Microsoft Excel database with advice from PD. Information was extracted on the source (first author, year, and title), study characteristics (including data sources, state/territory, time period, design), sample size including the number and proportion of Aboriginal and Torres Strait Islander participants, age range at diagnosis, cancer type, outcome measure (cause-specific survival, relative survival), statistical methods, covariates included in the statistical analyses and main findings relevant to the review question and objectives. Estimates for the Aboriginal & Torres Strait Islander (compared to other Australians) effect were extracted from both the unadjusted (where reported) and the fully adjusted survival model including all covariates. For each study, the data extract was independently checked against the original source by a second reviewer (PD).

If unadjusted survival estimates by Aboriginal and Torres Strait Islander status were not reported in a study, the corresponding author was contacted *via* email. While most of the authors contacted responded, many (nine studies) were not able to provide us with requested estimates primarily due to the dataset being no longer accessible.

### 2.10 Critical appraisal

Evaluating the risk of bias provides an indication of the quality of evidence that each study provides. Two reviewers PD and VMH independently assessed the risk of bias for a random selection of 18 of all eligible studies using the Joanna Briggs Institute critical appraisal checklist for cohort studies (**Supplementary Materials Table S1.2**) (27). Critical appraisal for the remaining studies was done by PD. The Joanna Briggs Institute tool has 11 domains regarding selection bias (1 item), confounding (2 items), validity of method used to measure exposure (1 item) and whether it was ascertained similarly for both exposed and unexposed groups (1 item) and outcome (2 items), follow-up time (3 items) and statistical methods (1 item). For each study, these items were answered with ‘yes’, ‘no’, ‘unclear’ or ‘not applicable’.

The following criteria were then used to determine the overall risk of bias for each study: low if at least 70% of all answers were yes (8≤ score ≤11), moderate if 50 to 69% questions were yes (6 ≤ score ≤7) and high if less than 50% of answers were yes (cohort 0 ≤ score ≤5). Studies were not excluded based on their quality score.

### 2.11 Presentation of results

Due to the variability across studies in time periods, statistical methodology, survival measures, covariates included and proportions of Aboriginal and Torres Strait Islander peoples within the study cohort, we have purposefully interpreted any general patterns cautiously and in a narrative format complemented by tables and figures. The focus is on whether there was evidence of disparities in cancer survival for Aboriginal and Torres Strait Islander peoples based on the unadjusted (where reported) and adjusted analyses, along with identifying those variables that explained some of the observed disparities. Results are presented initially for all cancers combined and then separately for different cancer types. Some studies are repeated across multiple tables.

The wide heterogeneity in methods across the included studies meant that no meta-analyses were conducted.

All analyses were performed with Stata/SE version 16 (RRID: SCR_012763). The Prisma diagram was created with PRISMA2020 package (28).

## 3 Results

### 3.1 Study selection

**Figure 1:**
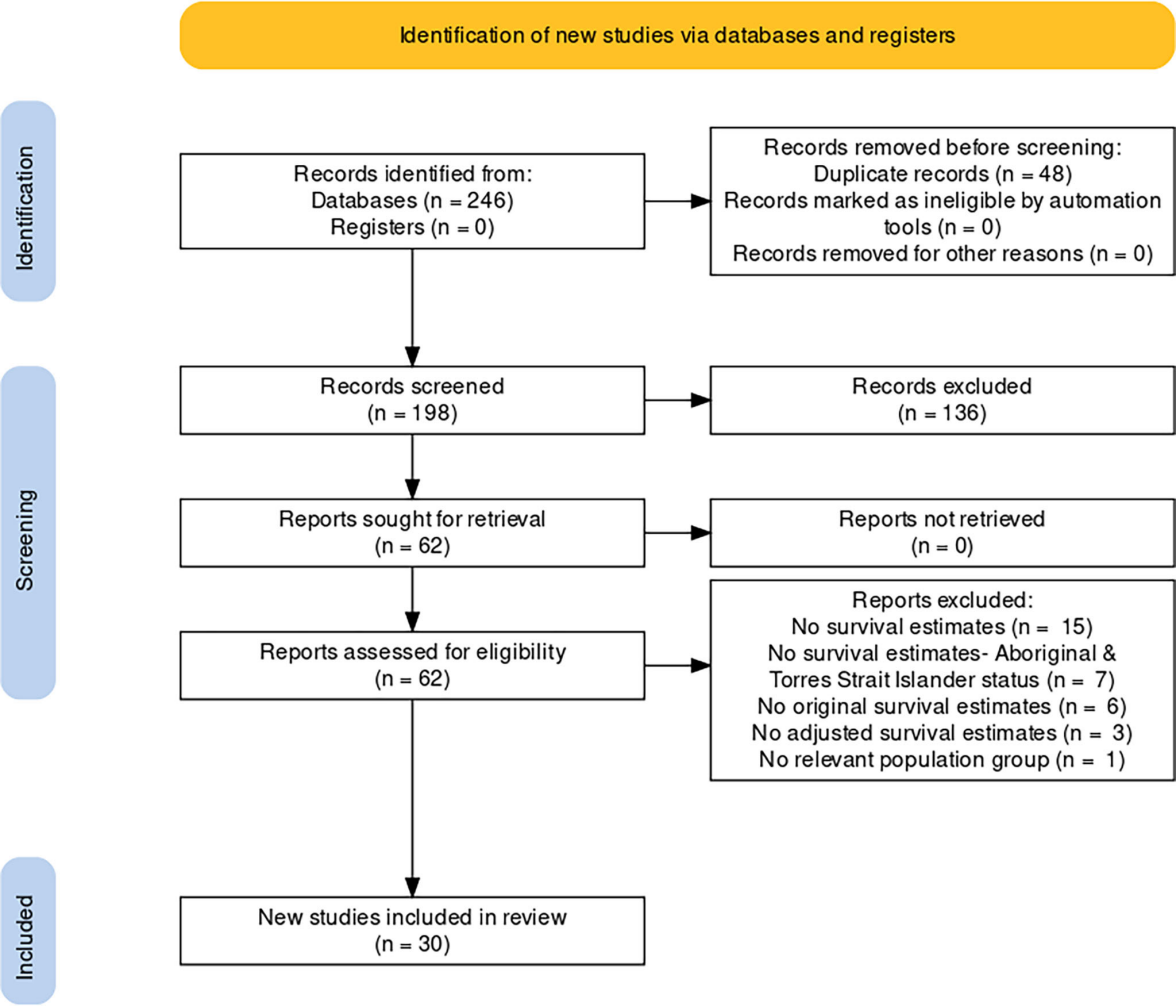
PRISMA 2020 flowchart for study selection.

Systematic searches identified 246 articles (**Figure 1**) of which, following title and abstract screening, 62 were selected for full-text screening. Thirty articles were retained for the review. The majority of the 32 excluded articles either did not report survival estimates or did not report them by Aboriginal and Torres Strait Islander status (**Supplementary Materials Table S1.3**).

### 3.2 Study characteristics

**Figure 2:**
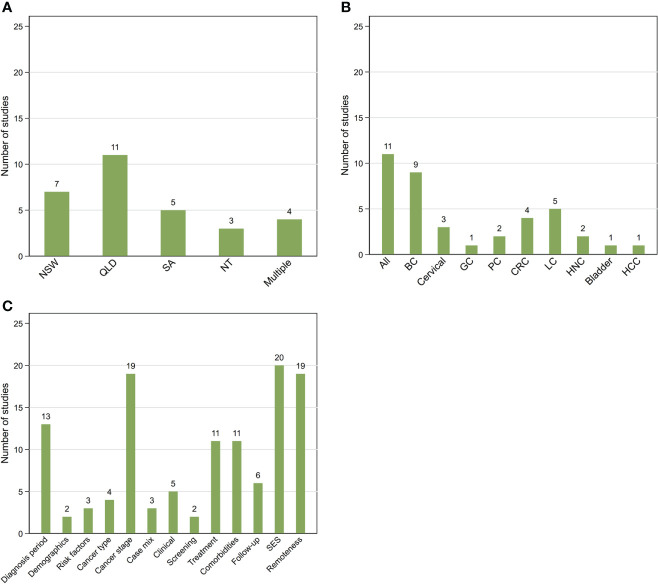
Summary of 30 included studies by location **(A)**, cancer type **(B)** and additional covariates **(C)** other than Aboriginal and Torres Strait Islander status, sex, and age included in survival models. NSW, New South Wales; QLD, Queensland; SA, South Australia; NT, Northern Territory; Multiple More than one state/territory **(A)**; BC, female breast cancer; GC, gynecological cancers; CRC, colorectal cancer; LC, lung cancer; HNC, head and neck cancers; HCC, hepatocellular carcinoma **(B)**; SES, socio-economic status.

All 30 included articles were observational cohort studies using data sourced from population-based cancer registries. Sixteen of these studies also linked the cancer registry data to other administrative hospital, treatment, and screening datasets. The majority (87%) were state based with only four studies assessing data from multiple states/territories in Australia (**Table 1**; **Figure 2A**). All but one study (43) included all adults diagnosed with cancer during study period. The time periods covered by the studies varied widely (range 1977 to 2017).

**Table 1:** Key characteristics of included studies.

Author, year	State/territory	Cancer type	Sample size (n)	Aboriginal and Torres Strait Islander n (% of sample)
Baade et al., 2016 (3)	QLD	All invasive	214,783	3,168 (1.5)
Banham et al., 2017 (29)	SA	All invasive	220,184	950 (0.4)
Chong & Roder, 2010 (30)	SA	All invasive	16,470	671 (4.1)
Condon et al., 2014 (4)	Semi-national^1^	All invasive	1,235,592	7,019 (0.6)
Cramb et al., 2012 (31)	QLD	All invasive	150,059	1,819 (1.2)
He et al., 2017 (32)	NT	All invasive	9,595	1,789 (18.6)
Morrell et al., 2012 (33)	NSW	All invasive	NR	2,604 (not known)
Peng & Baade, 2021 (6)	QLD	All invasive	217,819	3,987 (1.8)
Pule et al., 2018 (34)	SA	All invasive	1,554	777 (50.0)
Tervonen et al., 2016 (35)	NSW	All invasive	264,219	2,175 (0.8)
Tervonen et al., 2017 (36)	NSW	All invasive	301,356	2,517 (0.8)
Banham et al., 2019 (37)	SA	Female breast	154	77 (50.0)
Condon et al., 2016 (5)	NT	Female breast	1,283	196 (15.3)
Dasgupta et al., 2012 (38)	QLD	Female breast	18,568	202 (1.1)
Hsieh et al., 2016 (39)	QLD	Female breast	9,741	90 (0.9)
Moore et al., 2016a (40)	QLD	Female breast	215	110 (51.2)
Roder et al., 2012 (41)	National^2^	Female breast	5,366,983	36,204 (0.7)
Supramaniam et al., 2014 (42)	NSW	Female breast	27,850	288 (1.1)
Tervonen et al., 2017 (36)	NSW	Female breast	37,266	331 (0.9)
Youlden et al., 2020 (43)	QLD	Female breast	2,337	50 (2.1)
Condon et al., 2016 (5)	NT	Colorectal	1,104	110 (10.1)
Moore et al., 2016b (44)	QLD	Colorectal	165	80 (48.5)
Tervonen et al., 2017 (36)	NSW	Colorectal	40,288	289 (0.7)
Weir et al., 2016 (45)	NSW	Colorectal	29,777	298 (1.1)
Basnayake et al., 2021 (46)	NT	Lung	317	91 (28.7)
Condon et al., 2016 (5)	NT	Lung	1,256	359 (28.6)
Coory et al., 2008 (47)	QLD	Lung	310	158 (51.1)
Tervonen et al., 2017 (36)	NSW	Lung	27,302	392 (1.4)
Gibberd et al., 2016 (48)	NSW	NSCLC	20,154	341 (1.7)
Condon et al., 2016 (5)	NT	Cervical	233	86 (36.9)
Diaz et al., 2018 (49)	Semi-national^3^	Cervical	4,467	198 (4.4)
Diaz et al., 2015 (50)	QLD	Cervical	105	56 (53.3)
Diaz et al., 2015 (50)	QLD	Gynecological	257	137 (53.3)
Rodger et al., 2015 (51)	NSW	Prostate	35,214	259 (0.7)
Tervonen et al., 2017 (36)	NSW	Prostate	48,071	226 (0.5)
Luke et al., 2010 (52)	SA	Bladder	4,114	Not shown^4^
Condon et al., 2016 (5)	NT	Head and Neck	569	196 (34.4)
Moore et al., 2011 (53)	QLD	Head and Neck	129	67 (51.9)
Wigg et al., 2021^3^ (54)	QLD, SA, NT	HCC	3,816	229 (5.9)

ACT, Australian Capital Territory; HCC, hepatocellular carcinoma; NSCLC, non-small cell lung cancer; NSW, New South Wales; NT, Northern Territory; QLD, Queensland; SA, South Australia; WA, Western Australia.

1. All states/territories except Tasmania and Victoria.

2. All states/territories except ACT.

3. All states/territories except Tasmania and ACT.

4. Cell counts where number is less than twenty have been suppressed to protect confidentiality. These suppressions are denoted by “not shown”.

Ten studies reported survival outcomes for all invasive cancers combined, 18 for one and two for multiple cancer types (**Table 1**). The most analysed cancer type was female breast cancer (n=9) followed by lung cancer (n=5) (**Figure 2B**).

The number of Aboriginal and Torres Strait Islander peoples in included studies varied widely (range: <10 to 36,024) (**Table 1**). In terms of overall percentages, Aboriginal and Torres Strait Islander peoples comprised around 1% of the overall cohort for nine out of 29 studies (where this could be calculated), 2-10% (n=7), 10-30% (n=3), ≤0.5% (n=2) and around 50% in eight remaining studies.

All included studies controlled or adjusted for age and, where relevant, sex, while around three-quarters (n=23) also included area-level socio-economic status (SES) and/or remoteness based on residential address at time of cancer diagnosis as covariates. Nineteen studies also adjusted for some measure of stage at diagnosis while about half adjusted for diagnostic time-period. However, only those studies that used linked cancer registry and hospital data were able to adjust for treatment and clinical variables in the statistical analysis (**Table 2**; **Figure 2C**).

**Table 2:** Summary of included covariates other than age or sex for each of 30 included studies.

Author, year	Diagnosis period	Demographics^1^	Risk factors^2^	Cancer type	Stage^3^	Case-mix^4^	Clinical^5^	Screen status^6^	Treatment^7^	Comorbidities	Years since diagnosis	SES^8^	Remoteness^9^
Baade et al., 2016 (3)	N	N	N	N	N	N	N	N	N	N	N	N	N
Banham et al., 2017 (29)	N	N	N	N	Y	N	N	N	N	N	N	N	Y
Banham et al., 2019 (37)	N	N	N	N	Y	N	N	Y	Y	N	N	N	N
Basnayake et al., 2021 (46)	N	N	Y	N	Y	N	Y	N	Y	Y	N	Y	Y
Chong & Roder, 2010 (30)	Y	N	N	N	N	Y	N	N	N	N	N	N	N
Condon et al., 2014 (4)	N	N	N	Y	N	N	N	N	N	N	Y	N	N
Condon et al., 2016 (5)	Y	N	N	N	N	N	N	N	N	N	Y	N	N
Coory et al., 2008 (47)	N	N	N	N	Y	N	Y	N	Y	Y	N	Y	Y
Cramb et al., 2012 (31)	N	N	N	N	N	Y	N	N	N	N	Y	Y	Y
Dasgupta et al., 2012 (38)	Y	Y	N	N	Y	N	N	N	N	N	Y	Y	Y
Diaz et al., 2015 (50)	N	N	N	N	Y	N	N	N	Y	N	N	N	N
Diaz et al., 2018 (49)	N	N	N	N	N	N	Y	N	N	Y	N	Y	N
Gibberd et al., 2016 (48)	Y	N	Y	N	Y	N	N	N	Y	Y	N	Y	Y
He et al., 2017 (32)	N	N	N	Y	N	N	N	N	N	N	Y	N	N
Hsieh et al., 2016 (39)	N	Y	N	N	Y	N	N	Y	N	N	Y	Y	Y
Luke et al., 2010 (52)	N	N	N	N	N	N	Y	N	N	N	N	N	Y
Moore et al., 2011 (53)	N	N	N	N	Y	N	N	N	Y	Y	N	Y	N
Moore et al., 2016a (40)	N	N	N	N	Y	N	N	N	Y	Y	N	Y	N
Moore et al., 2016b (44)	N	N	N	N	Y	N	N	N	Y	Y	N	Y	Y
Morrell et al., 2012 (33)	Y	N	N	N	Y	N	N	N	N	N	N	N	N
Peng & Baade, 2021 (6)	N	N	N	N	N	Y	N	N	N	N	N	Y	Y
Pule et al., 2018 (34)	N	N	N	N	Y	N	N	N	N	N	N	Y	Y
Roder et al., 2012 (41)	Y	N	N	N	N	N	N	N	N	N	N	Y	Y
Rodger et al., 2015 (51)	Y	N	N	N	Y	N	N	N	Y	Y	N	Y	Y
Supramaniam et al., 2014 (42)	Y	N	N	N	Y	N	N	N	Y	Y	N	Y	Y
Tervonen et al., 2016 (35)	Y	N	N	Y	Y	N	N	N	N	N	N	Y	Y
Tervonen et al., 2017 (36)	Y	N	N	N	Y	N	N	N	N	N	N	Y	Y
Weir et al., 2016 (45)	Y	N	N	Y	Y	N	N	N	N	Y	N	Y	Y
Wigg et al., 2021 (54)	Y	N	Y	N	N	N	N	N	Y	Y	N	Y	Y
Youlden et al., 2020 (43)	Y	N	N	N	Y	N	Y	N	N	N	N	Y	Y

N, No; N/A, not applicable; SES, socio-economic status; Y, Yes.

1. Demographics are marital status or occupation.

2. Lifestyle risk factors such as smoking, obesity, alcohol status.

3. Stage or spread of disease at diagnosis.

4. Broad cancer group.

5. Clinical factors are cancer histology or morphology.

6. Breast-screening status.

7. Treatment-related factors such as receipt of surgery or curative therapies.

8. Area-level socio-economic status for residential location at time of diagnosis as measured by one of four Socio-Economic Indexes for areas in Australia from the Australian Bureau of Statistics.

9. Remoteness for residential location at time of diagnosis as measured by remoteness classification for Australia from the Australian Bureau of Statistics.

### 3.3 Outcome measures

**Figure 3:**
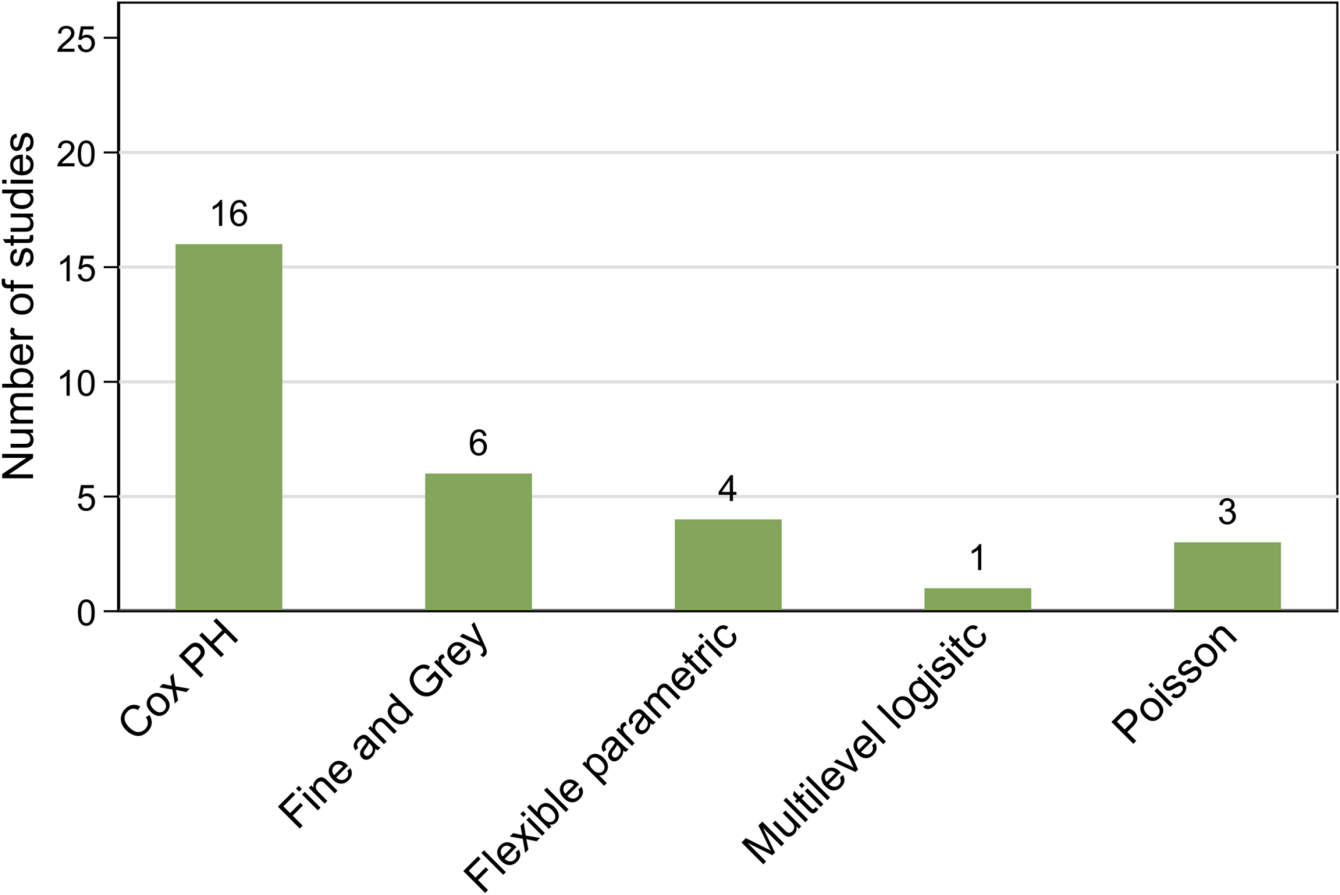
Summary of included studies by statistical model.

The majority (n=27) of studies reported cause-specific survival while three reported relative survival (4, 5, 39). Most studies focused on short to medium-term survival while seven did not specifically provide this information about length of follow-up (34–36, 39, 40, 44, 47). A variety of statistical models were used to quantify the survival differential by Aboriginal and Torres Strait Islander status and suggest associative factors (**Figure 3**). Hence the reported survival measures also varied although the majority reported either hazard ratios (HR), sub-hazard ratios (SHR) or excess hazard ratios (EHR) with corresponding standard errors or 95% confidence intervals (CI).

Fourteen studies reported survival differentials by Aboriginal and Torres Strait Islander status from both unadjusted and adjusted analyses. We were able to obtain the unadjusted survival estimate for Aboriginal and Torres Strait Islander peoples for a further five studies (i.e. published manuscripts) through personal communication with the study authors (3, 6, 31, 38, 43).

### 3.4 Risk of bias

Around three-quarters (n=22) of 30 studies were deemed to have a low risk of bias with scores of eight to nine for 11 domains of risk of bias tool (**Supplementary Materials Table S1.4**) and eight were classified as moderate (scores of 6 to 7). All studies were based on a reliable objective data source (cancer registries), assessed vital status through routine linkage of registry records to national death records and identified and controlled for at least age and sex (except for sex-specific cancer types). One noted cause of bias was the lack of information on follow-up in some studies. One potential source of bias, though not measured by the risk of bias tool, is the possible incomplete ascertainment of Aboriginal and Torres Strait Islander status by cancer registries, particularly for earlier publications.

### 3.5 Key findings

Studies are summarised below (**Table 3**, **Supplementary materials Tables S1.5-S1.9**).

**Table 3:** Summary of Aboriginal and Torres Strait Islander survival disparity for included studies by cancer type.

		Poorer survival: based on results from	
Author, year	Cancer type	Unadjusted model	Adjusted model^1^
Baade et al., 2016 (3)	All invasive	Not reported	Aboriginal and Torres-Strait Islander
Banham et al., 2017 (29)	All invasive	Not reported	Aboriginal and Torres-Strait Islander (s, r)
Chong & Roder et al, 2010 (30)	All invasive	Aboriginal and Torres-Strait Islander	Aboriginal and Torres-Strait Islander (p, r, m)
Condon et al., 2014 (4)	All invasive	Not reported	Aboriginal and Torres-Strait Islander^2^
Cramb et al., 2012 (31)	All invasive	Not reported	Aboriginal and Torres-Strait Islander^2^ (d, r, m)
He et al., 2017 (32)	All invasive	Not reported	Aboriginal and Torres-Strait Islander^2^ (m)
Morrell et al., 2012 (33)	All invasive	Not reported	Aboriginal and Torres-Strait Islander (s)
Peng & Baade, 2021 (6)	All invasive	Aboriginal and Torres-Strait Islander	Aboriginal and Torres-Strait Islander (d, r, m)
Pule et al., 2018 (34)	All invasive	Aboriginal and Torres-Strait Islander	Aboriginal and Torres-Strait Islander (s, d, r)
Tervonen et al., 2016 (35)	All invasive	Aboriginal and Torres-Strait Islander	Aboriginal and Torres-Strait Islander (p, d, r, m)
Tervonen et al., 2017 (36)	All invasive	Aboriginal and Torres-Strait Islander	Aboriginal and Torres-Strait Islander (s, d, r, p)
Banham et al., 2019 (37)	Female breast	Not reported	Aboriginal and Torres-Strait Islander (s, t)
Condon et al., 2016 (5)	Female breast	Not reported	Aboriginal and Torres-Strait Islander (p)
Dasgupta et al., 2012 (38)	Female breast	Not reported	Aboriginal and Torres-Strait Islander (s, d, r)
Hsieh et al., 2016 (39)	Female breast	Not reported	Aboriginal and Torres-Strait Islander (s, d, r)
Moore et al., 2016a (40)	Female breast	Aboriginal and Torres-Strait Islander	No difference (s, d, c, t)
Roder et al., 2012 (41)	Female breast	Not reported	Aboriginal and Torres-Strait Islander (d, r, p)
Supramaniam et al., 2014 (42)	Female breast	Aboriginal and Torres-Strait Islander	No difference (s, d, c, t, p)
Tervonen et al., 2017 (36)	Female breast	Not reported	Aboriginal and Torres-Strait Islander (s, d, r, p)
Youlden et al., 2020 (43)	Female breast	Aboriginal and Torres-Strait Islander	Aboriginal and Torres-Strait Islander (s, r, d, p)
Condon et al., 2016 (5)	Colorectal	Not reported	Aboriginal and Torres-Strait Islander (p)
Moore et al., 2016b (44)	Colorectal	No reported	No difference (s, r, d, c, t)
Tervonen et al., 2017 (36)	Colorectal	Not reported	Aboriginal and Torres-Strait Islander (s, d, r, p)
Weir et al., 2016 (45)	Colorectal	Aboriginal and Torres-Strait Islander	Aboriginal and Torres-Strait Islander (s, r, d, p, c, m)
Basnayake et al., 2021 (46)	Lung	Aboriginal and Torres-Strait Islander	No difference (s, r, d, h, t, c, f)
Condon et al., 2016 (5)	Lung	Not reported	Aboriginal and Torres-Strait Islander (p)
Coory et al., 2008 (47)	Lung	Aboriginal and Torres-Strait Islander	No difference (s, r, d, h, t, c)
Tervonen et al., 2017 (36)	Lung	Not reported	Aboriginal and Torres-Strait Islander (s, d, r, p)
Gibberd et al., 2016 (48)	NSCLC	Not reported	Aboriginal and Torres-Strait Islander (s, d, r, p, c, t, f)
Condon et al., 2016 (5)	Cervical	Not reported	Aboriginal and Torres-Strait Islander (p)
Diaz et al., 2018 (49)	Cervical	Not reported	Aboriginal and Torres-Strait Islander (d, c, h)
Diaz et al., 2015 (50)	Cervical	Aboriginal and Torres-Strait Islander	No difference (s, t)
Diaz et al., 2015 (50)	Gynecological	Aboriginal and Torres-Strait Islander	No difference (s, t)
Rodger et al., 2015 (51)	Prostate	Aboriginal and Torres-Strait Islander	Aboriginal and Torres-Strait Islander (s, d, r, p, c, t)
Tervonen et al., 2017 (36)	Prostate	Not reported	Aboriginal and Torres-Strait Islander (s, d, r, p)
Luke et al., 2010 (52)	Bladder	Not reported	Aboriginal and Torres-Strait Islander (h, r)
Condon et al., 2016 (5)	Head and Neck	Not reported	Aboriginal and Torres-Strait Islander (p)
Moore et al., 2011 (53)	Head and Neck	Aboriginal and Torres-Strait Islander	No difference (s, d, c, t)
Wigg et al., 2021^3^ (54)	HCC	Aboriginal and Torres-Strait Islander	No difference (r, d, c, t, f)

ACT, Australian Capital Territory; HCC, hepatocellular carcinoma; NSCLC, non-small cell lung cancer; NSW, New South Wales; NT, Northern Territory; QLD, Queensland; SA, South Australia; WA, Western Australia.

1. All adjusted for age and for sex unless gender-specific cancers.

(s) Also adjusted for some measure of spread of diagnosis, such as stage at diagnosis or spread of disease.

(t) Also adjusted for treatment-related factors.

(d) Also adjusted for area-disadvantage (socio-economic) of residential location at cancer diagnosis.

(c) Also adjusted for comorbidities.

(r) Also adjusted for remoteness of residential location at cancer diagnosis.

(p) Also adjusted for diagnostic year or time-period.

(m) Also adjusted for some measure of case-mix.

(h) Also adjusted for tumour histology or morphology.

(f) Also adjusted for at least one risk factor such as smoking.

2. Only at one-year after diagnosis.

#### 3.5.1 All invasive cancers

Overall, eleven studies explored disparities in cancer survival by Aboriginal and Torres Strait Islander status following diagnosis of any invasive cancer.

There was a consistent pattern of poorer survival among Aboriginal and Torres Strait Islander peoples for seven (3, 6, 30, 31, 34–36) state-based studies that reported unadjusted estimates (**Table 3**; **Supplementary materials Table S1.5**). Of the six studies that also reported estimates adjusted for various combinations of potential explanatory factors (other than age or sex), five found that the disparity reduced in magnitude after adjustment for remoteness, SES, and either cancer stage at diagnosis (34–36), or broad cancer group (6, 30) although it was still elevated and statistically significant. However, the remaining study, over an earlier time-period with less complete cultural identification, found that the adjusted differential was only elevated in magnitude and statistically significant up to one year after cancer diagnosis (31).

Of the four additional studies that reported no unadjusted survival differences, two found that the differential was only evident in the first year after diagnosis after adjusting for cancer type at the state (32) and national level (4). Finally, a statistically significant differential was seen after adjustment for stage in one study (33) or stage and remoteness in another study (29).

#### 3.5.2 Breast cancer

Aboriginal and Torres Strait Islander women had consistently poorer unadjusted breast cancer survival in four studies (38, 40, 42, 43), although the differential was not significant in one study (40) (**Table 3**; **Supplementary Materials Table S1.6**). Two of these studies also reported that this disparity remained elevated and statistical significant after adjustment for remoteness, SES, and tumour characteristics (38, 43) whereas two others found no evidence for a survival differential after additional adjustment for comorbidities and treatment (40, 42).

Of five other studies that only reported adjusted differentials, one found that Aboriginal and Torres Strait Islander women had significantly poorer breast cancer survival after adjustment for years since diagnosis (5). This differential was also evident after adjustment for cancer stage, screening history and treatment in one (37) or for remoteness, socio-demographics, stage, and detection method (screen versus symptomatic) in another state-level study (39). One study reported similar patterns after adjustment for remoteness, SES, and stage (36) while another large-scale national study reported a significant survival disparity after adjustment for remoteness and SES (41).

#### 3.5.3 Colorectal cancer

One study reported that the significantly poorer unadjusted colorectal cancer survival for Aboriginal and Torres Strait Islanders peoples was reduced after adjustment for remoteness, SES, comorbidities, stage, and cancer site to become non-significant although still elevated for non-surgically treated patients, whereas the corresponding differential remained for surgically treated patients (45) (**Table 3**, **Supplementary Materials Table S1.7**). Another small-scale study found no evidence of a survival disparity both before and after adjusting for remoteness, SES, stage, comorbidities, or treatment, although relatively small numbers of Aboriginal and Torres Strait Islander cases especially in some of the strata may have impacted the statistical power to assess differences (44). Two further studies did not report unadjusted estimates; however, a survival disparity was evident after adjustment for years since diagnosis (5) or remoteness, SES, and stage (36).

#### 3.5.4 Lung cancer

Two studies (46, 47) found that the observed (unadjusted) poorer lung cancer survival for Aboriginal and Torres Strait Islanders peoples was attenuated to become statistically non-significant after adjustment for remoteness, SES, stage at diagnosis, comorbidities, treatment and, for one study only (46), smoking status (**Table 3**; **Supplementary Materials Table S1.7**). Another earlier study while not reporting unadjusted estimates, however found a survival differential after adjustment for these covariates (48). Finally two more studies (no unadjusted estimates) reported Aboriginal and Torres Strait Islander disparities in lung cancer survival after adjusting for years since diagnosis (5) or diagnosis year, remoteness, SES, and stage (36).

#### 3.5.5 Cervical cancer (females)

There were two included studies that only reported adjusted survival differential for cervical cancer. Of these, one (state-level) reported significantly poorer survival among Aboriginal and Torres Strait Islander women after adjustment for years since diagnosis (5). Another semi-national study found a significant differential after adjustment for SES, tumour histology and comorbidities (49) (**Table 3**; **Supplementary Materials Table S1.8**). However, another (state-based) study reported that the differential (50) was no longer elevated or significant after adjustment for stage and treatment. In addition, this study also found that the observed survival differential for female gynecological cancers combined (i.e., cervical, uterine, ovarian, other) (50) reduced and became non-significant after adjustment for stage and treatment.

#### 3.5.6 Prostate cancer (males)

For prostate cancer, one study found that the significantly poorer unadjusted survival for Aboriginal and Torres Strait Islander men remained after adjustment for remoteness, SES, stage, comorbidities, and treatment, although the magnitude of the disparity reduced (51). Another study (no unadjusted estimates) also reported a statistically significant survival differential after adjustment for remoteness, SES, and stage (36) (**Table 3**; **Supplementary Materials Table S1.8**).

#### 3.5.7 Other cancer types

One study reported a statistically significant Aboriginal and Torres Strait Islander disparity in bladder cancer survival after adjustment for remoteness and tumour histology (no unadjusted estimates) (52) (**Table 3**; **Supplementary Materials Table S1.9**). Of two studies on survival disparities for head and neck cancers, one found that the unadjusted differential reduced after adjustment for SES, cancer stage, comorbidities, and curative treatment to become statistically non-significant although it was still elevated in magnitude (53) (**Table 3**, **Supplementary materials Table S1.9**). However, a survival disparity was evident after adjustment for follow-up time (no unadjusted estimates) in another study (5). Finally, the significantly poorer crude survival for Aboriginal and Torres Strait Islander peoples with hepatocellular carcinoma (HCC) was reduced after adjustment for remoteness, comorbidities, and curative treatment, so that the still elevated differential was no longer significant (54) (**Table 3**; **Supplementary Materials Table S1.9**).

## 4 Discussion

While cancer survival among Aboriginal and Torres Strait Islander peoples has improved over time, we found that Aboriginal and Torres Strait Islander cancer patients consistently experienced poorer survival than other Australians. However, the evidence was inconsistent regarding whether factors such as residential remoteness and SES, more advanced stage at diagnosis, greater comorbidities, and variations in treatment at least partially explained this disparity. A significant survival disparity remained after adjusting for these factors, particularly for all cancers combined and colorectal cancer, indicating that other, unmeasured factors affect the survival of Aboriginal and Torres Strait Islander cancer patients.

There is consensus in the published literature that additional factors occurring within and outside the cancer care pathway can explain at least some of the observed survival disparities (16, 17, 55, 56) These include logistical, social, environmental, health system and cultural such as systemic discrimination, perceptions about cancer, communication barriers, lower health literacy and lack of culturally appropriate care (2, 15–17, 55). International studies have consistently reported that the underlying causes for the cancer survival disparities among Indigenous peoples worldwide are complex and multifactorial that defy easy solutions (16, 17). It is likely, even in this age of “Big Data”, that quantitative data sources as used by the studies in this review may not provide the necessary information to fully identify the underlying reasons for the poorer survival for Aboriginal and Torres Strait Islander peoples diagnosed with cancer (45). This is particularly so in Australia since administrative data collections, including population-based cancer registries, do not routinely collect information on measures of individual-level socio-demographics, lifestyle or treatment-related factors including those related to decision-making and completion. More comprehensive, mixed methods research studies, such as the Cancer Data and Aboriginal Disparities Project (CanDAD), are likely necessary, in that they combine data linkage efforts (quantitative) with additional narrative reports (qualitative) to incorporate the experiences of Aboriginal and Torres Strait Islander cancer patients with cancer and the health system, as well as clinicians, family members and carers (57, 58). It is, however, also important to obtain the corresponding information from other Australians, including those of other cultural or migrant backgrounds, to better understand the differences in perceptions and behaviors from the perspective of various population subgroups that could help explain the survival disparities.

Improvements in, and expanded access to, culturally-sensitive and safe health care for all Australians will require changes at the service provider or health system level as well as the community or individual level, and to have the support of the community and senior health policy decision makers (57, 59). In practice, these could include increasing the number of Aboriginal and Torres Strait Islander health care workers, increasing the coverage of Indigenous-specific primary health care centres (10), and providing dedicated care coordination that is specifically designed to meet the needs of Aboriginal and Torres Strait Islander cancer patients and their families and carers in navigating the health care system (59). The provision of information that is appropriate and easily understood, and availability of suitable support services tailored to the needs of Aboriginal and Torres Strait Islander cancer patients is also important (59–61).

### 4.1 Strengths and limitations

This is the first systematic review to summarise contemporary published evidence for key factors explaining the survival disparity between Aboriginal and Torres Strait Islander peoples and other Australians following a cancer diagnosis. It was conducted according to established PRISMA guidelines, with multiple databases being searched with complex queries. All included articles were critically appraised for risk of bias. While this review did not consider the grey literature, an additional search using Google Scholar (62) and Australian Indigenous Health*InfoNet* databases (23) did not identify any reports that reported adjusted cancer survival estimates by Aboriginal and Torres Strait Islander status.

Given the considerable heterogeneity among studies in terms of the included covariates, statistical methods, reported survival measures and time-period, the summary patterns should be interpreted with caution. In addition, the high proportion of state/territory level studies, and the variability in cultural identification methods and quality between jurisdictions (24) meant that comparing and generalising results to a national level was difficult. However, despite these limitations, there remained a clear finding that these quantitative data do not explain all the observed survival disparities.

The number of Aboriginal and Torres Strait Islander peoples in study populations also varied widely and was often small. While this could limit the statistical power of studies to detect meaningful survival differences, that most studies still reported a statistically significant effect highlights the importance of these disparities. In addition, any conclusions from these studies are limited to those who identified themselves through self-reporting as Aboriginal or Torres Strait Islander people on health care databases.

The limited number of studies for individual cancer types, made it difficult to reach conclusions for specific cancer types, and highlights the importance of a coordinated research program involving sufficient numbers of Aboriginal and Torres Strait Islander peoples diagnosed with a range of cancer types.

## 5 Conclusions

Findings from this review were suggestive of as-yet un-identified factors that contribute to the observed survival disparities faced by Aboriginal and Torres Strait Islander peoples diagnosed with cancer. Further research that engages with Aboriginal and Torres Strait Islander communities using novel mixed methods approaches to better understand key contributors to these disparities is essential. Consistent and reliable evidence about the underlying causes and understanding these from the perspectives of Aboriginal and Torres Strait Islander peoples is required to better inform resource allocation to drive evidence-based interventions and more effective cancer control strategies to ensure best possible cancer outcomes for this population.

## Data availability statement

The original contributions presented in the study are included in the article/**Supplementary Material**. Further inquiries can be directed to the corresponding author.

## Author contributions

The authors PB, PD, and VMH contributed to the conception and design of the study. PB coordinated the study; PD and VMH conducted the literature searches; PD and VMH acted as reviewers; PD wrote the first draft of the manuscript, PB contributed to the initial draft of the manuscript. All authors, PB, PD, JA, VMH and GG contributed to manuscript revision, read, and approved the final submitted version. Each author has participated sufficiently in the work and takes responsibility for appropriate portions of the content.

## Funding

This research did not receive any specific grant from funding agencies in the public, commercial, or not-for-profit sectors. GG was supported by National Health and Medical Research Council (NHMRC) Investigator Grant (#1176651). Funding bodies had no role in the study design, collection, analysis, and interpretation of data, writing of this article or the decision to submit this article for publication.

## Conflict of interest

The authors declare that the research was conducted in the absence of any commercial or financial relationships that could be construed as a potential conflict of interest.

## Publisher’s note

All claims expressed in this article are solely those of the authors and do not necessarily represent those of their affiliated organizations, or those of the publisher, the editors and the reviewers. Any product that may be evaluated in this article, or claim that may be made by its manufacturer, is not guaranteed or endorsed by the publisher.

## Supplementary material

Supplemental checklist Prisma checklist. File name: Supplementary checklist.pdf

Supplemental material Additional tables. File name: Supplementary material.pdf
