# Supplementary material for: Factors associated with cancer survival disparities among Aboriginal and Torres Strait Islander peoples compared with other Australians: A systematic review
Source: Front Oncol. 2022 Sep 15;12:968400. doi: 10.3389/fonc.2022.968400 (PMC9521397; doi:10.3389/fonc.2022.968400)
Supplement: Supplemental checklist — Prisma checklist. File name: Supplementary checklist.pdf [file DataSheet_1.pdf]

Supplementary file Additional tables

1 Table S1.1 Database-specific search strategies

Database	Search strategy[1,2]
PubMed	(((((("neoplasms"[MeSH Terms]) OR (invasive neoplasm[MeSH Terms])) OR (cancer[Title])) OR (neoplasm*[Title])) AND (("Australia"[MeSH Terms]) OR ("Australia/epidemiology"[MeSH Terms]))) AND ((((((population, indigenous[MeSH Terms]) OR (populations, indigenous[MeSH Terms])) OR (oceanic ancestry group[MeSH Terms])) OR (indig*[Title/Abstract])) OR (Aborigin*[Title/Abstract])) OR (Torres Strait Island*[Title/Abstract]))) AND (((("survival"[MeSH Terms]) OR ("mortality"[MeSH Terms])) OR ("survival rate"[MeSH Terms])) OR ("survival analysis"[MeSH Terms]))
Embase	('cancer'/exp OR cancer) AND 'Australia'/exp AND ('indigenous Australian'/exp OR 'indigenous Australian' OR 'indigenous health care'/exp OR 'indigenous health care/exp' OR 'aboriginal Australian'/exp) AND ('survival'/exp OR 'survival' OR 'cancer mortality'/exp OR 'cancer mortality')
CINAHL	((MH "Neoplasms+") OR "neoplasm" AND (MH "Australia+")) AND ((MH "Indigenous Peoples+") OR (MH "Health Services, Indigenous") OR (MH "Indigenous Health") OR AB indig* AND (MH "Survival") OR (MH "Survival Analysis+") OR (MH "Mortality+") OR AB "survival")
Web of Science	Aboriginal OR Torres Strait Islander* OR Indigenous (Topic) AND Australia (Topic) AND Cancer (Topic) AND Survival (Topic)
Australian Indigenous Health*InfoNet*	Survival and cancer ('title & abstract')

1. All Electronic databases searched: (2010- 4 May 2022).
2. All search queries were conducted in a stepwise manner by breaking down each question into key concepts. For each element, alternative terms were used to cover all possible synonyms for that component. Finally, the individual search queries were combined to create the final search query using BOOLEAN operators such as "AND" or "OR".

2 Table S1.2 The Joanna Briggs Institute (JBI) critical appraisal tool for cohort studies used for risk of bias assessment of included studies

**Major Components**	**Response options**			
1. Were the two groups similar and recruited from the same population?	Yes	No	Unclear	Not applicable
2. Were the exposures measured similarly to assign people to both exposed and unexposed groups?	Yes	No	Unclear	Not applicable
3. Was the exposure measured in a valid and reliable way?	Yes	No	Unclear	Not applicable
4. Were confounding factors identified?	Yes	No	Unclear	Not applicable
5. Were strategies to deal with confounding factors stated?	Yes	No	Unclear	Not applicable
6. Were the groups/participants free of the outcome at the start of the study (or at the moment of exposure)?	Yes	No	Unclear	Not applicable
7. Were the outcomes measured in a valid and reliable way?	Yes	No	Unclear	Not applicable
8. Was the follow up time reported and sufficient to be long enough for outcomes to occur?	Yes	No	Unclear	Not applicable
9. Was follow up complete, and if not, were the reasons to loss to follow up described and explored?	Yes	No	Unclear	Not applicable
10. Were strategies to address incomplete follow up utilized?	Yes	No	Unclear	Not applicable
11. Was appropriate statistical analysis used?	Yes	No	Unclear	Not applicable

3 Table S1.3 Excluded studies with reasons for exclusion

Author, year	Reason for exclusion
AIHW, 2011 (1)	No survival estimates
AIHW, 2013 (2)	No survival estimates
AIHW, 2015 (3)	No survival estimates
AIHW, 2018 (4)	No survival estimates
Baade et al., 2010 (5)	No survival estimates
Banham et al., 2018 (6)	No survival estimates by Aboriginal & Torres Strait Islander status
Banham et al., 2019 (7)	No survival estimates by Aboriginal & Torres Strait Islander status
Barraclough et al., 2016 (8)	No survival estimates
Clark et al., 2015 (9)	No survival estimates by Aboriginal & Torres Strait Islander status
Condon et al., 2011 (10)	No original survival estimates
Davidson et al., 2013 (11)	No original survival estimates
Davies et al., 2021 (12)	No original survival estimates
Diaz et al., 2015 (13)	No original survival estimates
Fitzadam et al., 2021 (14)	No survival estimates
Gibberd et al., 2015 (15)	No survival estimates
Haggar et al., 2013 (16)	Not target population group
Ho et al., 2009 (17)	No adjusted survival estimate
Hopkins & Dolan, 2017 (18)	No survival estimates
Khan et al, 2020a (19)	No survival estimates
Khan et al, 2020b (20)	No adjusted survival estimate
Khan et al, 2021 (21)	No survival estimates
Luke et al, 2010 (22)	No survival estimates by Aboriginal & Torres Strait Islander status
Martin et al, 2009 (23)	No survival estimates by Aboriginal & Torres Strait Islander status
Parker et al., 2014 (24)	No survival estimates by Aboriginal & Torres Strait Islander status
Read & Hayes, 2019 (25)	No survival estimates
Read et al., 2020 (26)	No survival estimates
Roder et al., 2014 (27)	No survival estimates by Aboriginal & Torres Strait Islander status
Tan et al., 2016 (28)	No survival estimates
Tapia et al, 2017 (29)	No original survival estimates
Tervonen et al, 2019 (30)	No survival estimates
Tomita et al, 2016 (31)	No adjusted survival estimate
Withrow et al, 2016 (32)	No original survival estimates

4 Table S1.4 Results of critical appraisal for studies included in systematic review

	Critical appraisal questions[1]											Risk of bias [2,3]	
Author, year	Q1	Q2	Q3	Q4	Q5	Q6	Q7	Q8	Q9	Q10	Q11	**Total score**	**Category**
Baade et al., 2016 (33)	U	Y	Y	Y	Y	Y	Y	Y	Y	N/A	Y	9	**Low**
Banham et al., 2017 (34)	U	Y	Y	Y	Y	Y	Y	U	Y	N/A	Y	8	**Low**
Banham et al., 2019 (35)	U	Y	Y	Y	Y	Y	Y	U	Y	N/A	Y	8	**Low**
Basnayake et al., 2021 (36)	U	Y	Y	Y	Y	Y	Y	Y	Y	N/A	Y	9	**Low**
Chong & Roder, 2010 (37)	U	Y	Y	Y	Y	Y	Y	U	U	N/A	Y	7	**Moderate**
Condon et al., 2014 (38)	U	Y	Y	Y	Y	Y	Y	Y	Y	N/A	Y	9	**Low**
Condon et al., 2016 (39)	U	Y	Y	Y	Y	Y	Y	U	U	N/A	Y	7	**Moderate**
Coory et al., 2008 (40)	U	Y	Y	Y	Y	Y	Y	Y	Y	N/A	Y	9	**Low**
Cramb et al., 2012 (41)	U	Y	Y	Y	Y	Y	Y	Y	Y	N/A	Y	9	**Low**
Dasgupta et al., 2012 (42)	U	Y	Y	Y	Y	Y	Y	Y	Y	N/A	Y	9	**Low**
Diaz et al., 2015 (43)	Y	Y	Y	Y	Y	Y	Y	U	U	N/A	U	7	**Moderate**
Diaz et al., 2018 (44)	U	Y	Y	Y	Y	Y	Y	U	U	N/A	Y	7	**Moderate**
Gibberd et al., 2016 (45)	U	Y	Y	Y	Y	Y	Y	Y	Y	N/A	Y	9	**Low**
He et al., 2017 (46)	U	Y	Y	Y	Y	Y	Y	Y	Y	N/A	Y	9	**Low**
Hsieh et al., 2016 (47)	U	Y	Y	Y	Y	Y	Y	Y	Y	N/A	U	8	**Low**
Luke et al., 2010 (48)	U	Y	Y	Y	Y	Y	Y	U	U	N/A	Y	7	**Moderate**
Moore et al., 2011 (49)	U	Y	Y	Y	Y	Y	Y	U	U	N/A	Y	7	**Moderate**
Moore et al., 2016a (50)	Y	Y	Y	Y	Y	Y	Y	U	Y	N/A	Y	9	**Low**
Moore et al., 2016b (51)	U	Y	Y	Y	Y	Y	Y	Y	Y	N/A	Y	9	**Low**
Morrell et al., 2012 (52)	U	Y	Y	Y	Y	Y	U	U	U	N/A	Y	6	**Moderate**
Peng & Baade 2021 (53)	U	Y	Y	Y	Y	Y	Y	Y	Y		Y	9	**Low**
Pule et al., 2018 (54)	U	Y	Y	Y	Y	Y	Y	Y	Y	N/A	Y	9	**Low**
Roder et al., 2012 (55)	U	Y	Y	Y	Y	Y	Y	Y	U	N/A	Y	8	**Low**
Rodger et al., 2015 (56)	U	Y	Y	Y	Y	Y	Y	N	U	N/A	Y	7	**Moderate**
Supramaniam et al., 2014 (57)	U	Y	Y	Y	Y	Y	Y	Y	Y	N/A	Y	9	**Low**
Tervonen et al., 2016 (58)	U	Y	Y	Y	Y	Y	Y	Y	Y	N/A	Y	9	**Low**
Tervonen et al., 2017 (59)	U	Y	Y	Y	Y	Y	Y	Y	Y	N/A	Y	9	Low
Weir et al., 2016 (60)	U	Y	Y	Y	Y	Y	Y	Y	Y	N/A	Y	9	**Low**
Wigg et al., 2021 (61)	U	Y	Y	Y	Y	Y	Y	Y	Y	N/A	Y	9	**Low**
Youlden et al., 2020 (62)	U	Y	Y	Y	Y	Y	Y	Y	Y	N/A	Y	9	**Low**

Y=yes; N=no; U=unclear; N/A=not applicable.

1. Q. 1: Participants were appropriately sampled, Q. 2: Exposure clearly defined and described, Q. 3: Exposure measured in a valid and reliable way, Q. 4: Confounding factors identified, Q. 5: Confounding factors accounted for, Q. 6: Participants free of outcome at start of study, Q. 7: Outcome measured in a valid and reliable way, Q. 8: Follow up reported and sufficient, Q. 9: Follow up complete or reasons for loss described, Q. 10: Strategies to address incomplete follow up, Q. 11: Appropriate statistical analyses were used
2. Risk of bias based on Joanna Briggs Institute critical appraisal tools for cohort studies.
3. Risk of bias ranked as low risk of bias if at least 70% of all answers were yes (8≤ score ≤11), moderate risk if 50 to 69% questions were yes (:6 ≤ score ≤7) and high risk of bias if yes answers were below 50% (0 ≤ score ≤5).

5 Table S1.5 Characteristics of included studies on survival outcomes for all invasive cancers combined by Aboriginal and Torres Strait Islander status

Study	State/territory	Source	Design	Sample[1]	Period (follow-up)	Outcome	Analysis	Covariates	Unadjusted results	Adjusted results
Baade et al., 2016 (33)	QLD	CR	Cohort	214,783 (3,168 Aboriginal and Torres Strait Islander) aged 20-89 years	2007-2012 (end 2013)	5-year cause-specific survival	Flexible Parametric models	Age, sex	CSR 0.8 (0.7-0.9)[2,3]	CSR[3] 0.9 (0.8-0.9)
Banham et al., 2017 (34)	SA	CR and APDC	Matched cohort, data linkage	220,184 (950 Aboriginal and Torres Strait Islander)	1977-2010 (end 2011)	5-year cause-specific survival	Fine and Gray regression	Age, sex, remoteness, stage	N/R	SHR 1.6 (1.4-1.9)
Chong & Roder, 2010 (37)	SA	CR	Cohort	16,470(671 Aboriginal and Torres Strait Islander)	1997-2007 (end)	5-year cause-specific survival	Cox PH regression	Age, sex, diagnostic period, remoteness, prognostic index	HR 2.0 (1.8-2.2)	HR 1.4 (1.2-1.5)
Condon et al., 2014 (38)	Semi-national (except Tasmania, Victoria)	CR	Cohort	1,235,592 (7,019 Aboriginal and Torres Strait Islander)	2001-2005 (end 2007)	5-year relative survival	Poisson regression	Age, sex, cancer type, follow-up time	N/R	1 year follow-up: EHR 1.9 (1.8-2.0) 5-years 0.7 (0.3-1.4)
Cramb et al., 2012 (41)	QLD	CR	Cohort	150,059 (1,819 Aboriginal and Torres Strait Islander)	1997-2006 (end 2007)	5-year cause-specific survival	Cox PH regression	Age, sex, remoteness, SES, cancer group, follow-up time	HR 1.5 (1.4-1.6)[2]	1 year follow-up HR: 1.5 (1.4-1.6); 5-years 0.7 (0.4-1.1)
He et al., 2017 (46)	NT	CR	Cohort	9,595 (1,789 Aboriginal and Torres Strait Islander)	1991-2010 (end 2011)	5-year cause-specific survival	Cox PH regression	Age, sex, cancer type, follow-up time	N/R	1 year follow-up HR: 2.2 (2.0-2.4); 5-years 0. 8 (0.5-1.3)
Morrell et al., 2012 (52)	NSW	CR	Cohort	Overall size N/S (2,604 Aboriginal and Torres Strait Islander)	1999-2008	5-year cause-specific survival	Cox PH regression	Age, sex, diagnosis year, spread of disease	N/R	HR 1.6 (1.5-1.7)

Study	State/territory	Source	Design	Sample[1]	Period (follow-up)	Outcome	Analysis	Covariates	Unadjusted results	Adjusted results
Peng & Baade 2021 (53)	QLD	CR	Cohort	217,819 (3,987 Aboriginal and Torres Strait Islander), aged 15-89 years	2008-2017	5-year cause-specific survival	Flexible Parametric models	Age, sex, remoteness, SES, cancer group	HR 1.6 (1.5-1.7)[2]	HR 1.4 (1.3-1.5)
Pule et al., 2018 (54)	SA	CR and APDC	Cohort	1,554 (777 Aboriginal and Torres Strait Islander)	1990-2010	Cause-specific survival	Fine and Gray regression	Age, sex, remoteness, SES, stage	SHR 2.0 (1.7-2.2)	SHR 1.6 (1.3-1.9)
Tervonen et al., 2016 (58)	NSW	CR	Cohort	264,219 (2,175 Aboriginal and Torres Strait Islander)	2000-2008	Cause-specific survival	Fine and Gray regression	Age, sex, diagnosis year, remoteness, SES, stage, cancer type	Most disadvantaged SHR 1.5 (1.4-1.6);	Most disadvantaged SHR 1.3 (1.2-1.5)
Tervonen et al., 2017 (59)	NSW	CR	Cohort	301,356 (2,517 Aboriginal and Torres Strait Islander)	2000-2008	Cause-specific survival	Fine and Gray regression	Age, sex, diagnosis year, remoteness, SES, stage	SHR 1.5 (1.4-1.6)	SHR 1.4 (1.3-1.5)

ACT Australian Capital Territory, APDC Department of Health Admitted Patient Data Collection, CR Cancer Registry, EHR Excess Hazard Ratio, HR Hazard Ratio; N/R not reported, NSW New South Wales, NT Northern Territory, QLD Queensland, SA South Australia, PH Proportional Hazards, SES socio-economic status, SHR Sub-Hazard Ratio, WA Western Australia

1. Included all adults aged at least 15 years unless otherwise stated
2. Estimates obtained from corresponding author
3. CSR is the comparative survival ratio, defined as the ratio of the observed cause-specific survival (Aboriginal and Torres Strait Islanders) to the observed cause-specific survival (other Australians) A CSR estimate of <1 indicates poorer survival among Aboriginal and Torres Strait Islanders.

Table S1.6 Characteristics of included studies on female breast cancer survival outcomes by Aboriginal and Torres Strait Islander status

Study	State/territory	Source	Design	Sample (women aged ≥15 years)	Period (follow-up)	Outcome	Analysis	Covariates	Unadjusted results	Adjusted results
Banham et al., 2019 (35)	SA	CR, BreastScreen and APDC	Matched cohort, data linkage	154 (77 Aboriginal and Torres Strait Islander)	1990-2010 (end 2011)	5-year cause-specific survival	Fine and Gray regression	Age, stage, screening history, treatment	N/R	SHR 5.1 (1.2-21.7)
Condon et al., 2016 (39)	NT	CR	Cohort	1,283 (196 Aboriginal and Torres Strait Islander)	1991-2010 (end 2012)	5-year relative survival	Poisson regression	Age, sex, diagnostic period, follow-up time	N/R	EHR 3.1 (2.1-4.0)
Dasgupta et al., 2012 (42)	QLD	CR	Cohort	18,568 (202 Aboriginal and Torres Strait Islander) aged 30-79 years	1997-2006 (end 2007)	5-year cause-specific survival	Multilevel logistic regression	Age, remoteness, SES, diagnosis year, stage, occupation, marital status, follow-up time	HR 1.9 (1.3-2.7)[1]	HR 1.6 (1.1-2.1)
Hsieh et al., 2016 (47)	QLD	CR and BreastScreen	Cohort, data linkage	9,741 (90 Aboriginal and Torres Strait Islander) aged 40-89 years	1997-2007 (end 2008)	Relative survival	Poisson regression	Age, remoteness, SES, stage, occupation, detection method, follow-up time	N/R	RER 2.9 (1.5-4.7)
Moore et al., 2016a (50)	QLD	CR and hospital records	Matched cohort, data linkage	215 (110 Aboriginal and Torres Strait Islander)	1998-2004 (end 2006)	Cause-specific survival	Cox PH regression	Age, stage, comorbidities, treatment, SES	HR 1.9 (1.0-3.6)	HR 1.4 (0.7-2.8)
Roder et al., 2012 (55)	National (except ACT)	CR and BreastScreen	Cohort, data linkage	5,366,983 (36,204 Aboriginal and Torres Strait Islander)	1991-2006	5-year cause-specific survival	Cox PH regression	Age, remoteness, SES, diagnostic period	N/R	HR 1.7 (1.4-2.0)
Supramaniam et al., 2014 (57)	NSW	CR and APDC	Cohort, data linkage	27, 850 (288 Aboriginal and Torres Strait Islander) aged ≥18 years	2001-2007 (end 2008)	5-year cause-specific survival	Cox PH regression	Age, remoteness, SES, comorbidities, diagnosis year,	HR 1.7 (1.2-2.3)	HR 1.3 (0.9-1.8)

Study	State/territory	Source	Design	Sample (women aged ≥15 years)	Period (follow-up)	Outcome	Analysis	Covariates	Unadjusted results	Adjusted results
								spread of disease, surgical treatment		
Tervonen et al., 2017 (59)	NSW	CR	Cohort	37,266 (331 Aboriginal and Torres Strait Islander)	2000-2008	Cause-specific survival	Fine and Gray regression	Age, sex, diagnosis year, remoteness, SES, stage	N/R	SHR 1.6 (1.2-2.2)
Youlden et al., 2020 (62)	QLD	CR	Cohort	2,337 (50 Aboriginal and Torres Strait Islander) aged 15-39 years	1997-2014 (end 2016)	5-year cause-specific survival	Flexible Parametric models	Age, remoteness, SES, diagnosis year, stage, morphology	HR 3.6 (2.2-6.0)[1]	HR 2.7 (1.6-4.6)

ACT Australian Capital Territory, APDC Department of Health Admitted Patient Data Collection, CR Cancer Registry, EHR Excess Hazard Ratio, HR Hazard Ratio; N/R not reported, NSW New South Wales, NT Northern Territory, QLD Queensland, SA South Australia, PH Proportional Hazards, RER Relative Excess Risk, SES socio-economic status, SHR Sub-Hazard Ratio

1. Estimates obtained from corresponding author

Table S1.7 Characteristics of included studies on survival outcomes for colorectal or lung cancers by Aboriginal and Torres Strait Islander status

Study	State/territory	Source	Design	Sample[1]	Period (follow-up)	Outcome	Analysis	Covariates	Unadjusted results	Adjusted results
**Colorectal cancer**										
Condon et al., 2016 (39)	NT	CR	Cohort	1,104 (110 Aboriginal and Torres Strait Islander)	1991-2010 (end 2012)	5-year relative survival	Poisson regression	Age, sex, diagnostic period, follow-up time	N/R	EHR 2.2 (1.6-3.0)
Moore et al., 2016b (51)	QLD	CR and hospital records	Matched cohort, data linkage	165 (80 Aboriginal and Torres Strait Islander)	1998-2004 (end 2006)	Cause-specific survival	Cox PH regression	Age, sex, remoteness, SES, comorbidities, stage, treatment	HR 1.0 (0.6-1.7)	HR 1.3 (0.7-2.3)
Tervonen et al., 2017 (59)	NSW	CR	Cohort	40,288 (289 Aboriginal and Torres Strait Islander)	2000-2008	Cause-specific survival	Fine and Gray regression	Age, sex, diagnosis year, remoteness, SES, stage	N/R	SHR 1.6 (1.3-1.9)
Weir et al., 2016 (60)	NSW	CR and APDC	Cohort, data linkage	29,777 (278 Aboriginal and Torres Strait Islander)	2001-2007 (end 2008)	5-year cause-specific survival	Cox PH regression	Age, sex, remoteness, SES, diagnosis year, spread of disease, cancer site[2], comorbidities	HR 1.3 (1.1-1.6)	Surgery<br>HR 1.7 (1.3-2.1<br>No surgery<br>HR 1.1 (0.7-1.5)
**Lung cancer**										
Basnayake et al., 2021 (36)	NT	CR and hospital records	Cohort, data linkage	317 (91 Aboriginal and Torres Strait Islander)	2010-2014 (end 2017)	5-year cause-specific survival	Cox PH regression	Age, sex, remoteness, SES, stage, histology, comorbidities, treatment, smoking status, functional status	HR 1.4 (1.0-1.8)	HR 1.4 (1.0-2.1)
Condon et al., 2016 (39)	NT	CR	Cohort	1,256 (359 Aboriginal and Torres Strait Islander)	1991-2010 (end 2012)	5-year relative survival	Poisson regression	Age, sex, diagnostic period, follow-up time	N/R	EHR 1.4 (1.2-1.6)

Study	State/territory	Source	Design	Sample[1]	Period (follow-up)	Outcome	Analysis	Covariates	Unadjusted results	Adjusted results
Coory et al., 2008 (40)	QLD	CR and hospital records	Matched cohort, data linkage	310 (158 Aboriginal and Torres Strait Islander)	1996-2002 (end 2003)	Cause-specific survival	Cox PH regression	Age, sex, remoteness, SES, histology, stage, treatment, comorbidities	HR 1.5 (1.1-1.9)	HR 1.0 (0.8-1.4)
Tervonen et al., 2017 (59)	NSW	CR	Cohort	27,302 (392 Aboriginal and Torres Strait Islander)	2000-2008	Cause-specific survival	Fine and Gray regression	Age, sex, diagnosis year, remoteness, SES, stage	N/R	SHR 1.4 (1.2-1. 6)
Gibberd et al., 2016 (45)	NSW	CR and APDC	Cohort, data linkage	20,154 (341 Aboriginal) aged ≥18 years, NSCLC	2001-2007 (end 2008)	5-year cause-specific survival	Fine and Gray regression	Age, sex, remoteness, SES, spread of disease, diagnosis year, surgical treatment, comorbidities, smoking status	N/R	SHR 1.3 (1.1-1.5)

ACT Australian Capital Territory, APDC Department of Health Admitted Patient Data Collection, CR Cancer Registry, EHR Excess Hazard Ratio, HR Hazard Ratio; N/R not reported, NSW New South Wales, NT Northern Territory, QLD Queensland, SA South Australia, PH Proportional Hazards, SES socio-economic status, SHR Sub-Hazard Ratio

1. Included all adults aged at least 15 years unless otherwise stated
2. Site of colorectal cancer: colon, rectum, rectosigmoid)

8 **Table S1.8 Characteristics of included studies on survival outcomes for other gender specific cancers (cervical; gynaecological or prostate) by Aboriginal and Torres Strait Islander status**

Study	State/territory	Source	Design	Sample[1]	Period (follow-up)	Outcome	Analysis	Covariates	Unadjusted results	Adjusted results
						**Cervical cancer**				
Condon et al., 2016 (39)	NT	CR	Cohort	233 women (86 Aboriginal and Torres Strait Islander)	1991-2010 (end 2012)	5-year relative survival	Poisson regression	Age, diagnostic period, follow-up time	N/R	EHR 3.2 (2.0-5.2)
Diaz et al., 2018 (44)	Semi-national (except Tasmania, ACT)	CR and APDC	Cohort, data linkage	4,467 women (198 Aboriginal and Torres Strait Islander)	2003 (VIC 2007) -2012	5-year cause-specific survival	Flexible parametric models	Age, SES, histology, comorbidities,	N/R	HR 1.8 (1.4-2.3)
Diaz et al., 2015 (43)	QLD	CR and APDC	Matched cohort, data linkage	105 women (56 Aboriginal and Torres Strait Islander) aged ≥18 years	1998-2004	1 year cause-specific survival	Cox PH regression	Age, stage, treatment	HR 2.5 (1.1-5.9)	HR 1.0 (0.5-2.2)
						**Gynaecological cancers** (cervical, ovarian, uterine cancers, other gynaecological)				
Diaz et al., 2015 (43)	QLD	CR and APDC	Matched cohort, data linkage	257 women (137 Aboriginal and Torres Strait Islander) aged ≥18 years	1998-2004	1 year cause-specific survival	Cox PH regression	Age, stage, treatment	HR 1. 9 (1.1-3.4)	HR 1.2 (0.7-2.3)
						**Prostate cancer**				
Rodger et al., 2015 (56)	NSW	CR and APDC	Cohort, data linkage	35,214 men (259 Aboriginal and Torres Strait Islander), localised disease	2001-2007 (end 2008)	5-year cause-specific survival	Cox PH regression	Age, remoteness, SES, diagnosis year, spread of disease, comorbidities, surgical treatment	HR 1.8 (1.3-2.4)	HR 1.5 (1.1-2.0)
Tervonen et al., 2017 (59)	NSW	CR	Cohort	48,071 men (226 Aboriginal and Torres Strait Islander)	2000-2008	Cause-specific survival	Fine and Gray regression	Age, sex, diagnosis year, remoteness, SES, stage	N/R	SHR 1.9 (1.2-2.8)

ACT Australian Capital Territory, APDC Department of Health Admitted Patient Data Collection, CR Cancer Registry, EHR Excess Hazard Ratio, HR Hazard Ratio; N/R not reported, NSW New South Wales, NT Northern Territory, QLD Queensland, SA South Australia, PH Proportional Hazards, SES socio-economic status, SHR Sub-Hazard Ratio

1. Included all adults aged at least 15 years unless otherwise stated.

9 Table S1.9 Characteristics of included studies on survival outcomes for bladder, head and neck or liver cancers by Aboriginal and Torres Strait Islander status

Study	State/territory	Source	Design	Sample[1]	Period (follow-up)	Outcome	Analysis	Covariates	Unadjusted results	Adjusted results
					**Bladder cancer**					
Luke et al., 2010 (48)	SA	CR	Cohort	4,114 (<10 Aboriginal and Torres Strait Islander)	1980-2004	5-year cause-specific survival	Cox PH regression	Age, sex, remoteness, histology	N/R	HR 3.2 (1.3-7.7)
					**Head and neck cancers**					
Condon et al., 2016 (39)	NT	CR	Cohort	569 (196 Aboriginal and Torres Strait Islander)	1991-2010 (end 2012)	5-year relative survival	Poisson regression	Age, sex, diagnostic period, follow-up time	N/R	EHR 2.1 (1.6-2.7)
Moore et al., 2011 (49)	QLD	CR and hospital records	Matched cohort, data linkage	129 (67 Aboriginal and Torres Strait Islander), aged ≥18 years	1998-2004	5-year cause-specific survival	Cox PH regression	Age, sex, SES, stage, comorbidities, treatment (curative)	HR 2.5 (1.5-4.0)	HR 1.6 (0.9, 2.7)[4]
					**Hepatocellular carcinoma (HCC)**					
Wigg et al., 2021(61)	QLD, SA, NT	CR and APDC	Cohort, data linkage	3,816 (229 Aboriginal and Torres Strait Islander), aged ≥18 years, HCC	2000-2015 (NT) 2000-2017 (QLD) 2000-2018 (SA)	5-year cause-specific survival	Cox PH regression	Age, sex, remoteness, comorbidities, state, diagnostic period, treatment, HCC risk factors	HR 1.4 (1.2-1.7)	HR 1.2 (1.0-1.5)

ACT Australian Capital Territory, APDC Department of Health Admitted Patient Data Collection, CR Cancer Registry, EHR Excess Hazard Ratio, HR Hazard Ratio; N/R not reported, NSW New South Wales, NT Northern Territory, QLD Queensland, SA South Australia, PH Proportional Hazards, SES socio-economic status, SHR Sub-Hazard Ratio

1. Included all adults aged at least 15 years unless otherwise stated

10 References

1. Australian Institute of Health and Welfare. Cancer in Australia: Actual incidence and mortality data from 1982 to 2007 and projections to 2010. *Asia Pac J Clin Oncol* (2011) 7(4):325-38. doi: 10.1111/j.1743-7563.2011.01502.x.
2. Australian Institute of Health and Welfare. Cancer in Australia: Actual incidence data from 1991 to 2009 and mortality data from 1991 to 2010 with projections to 2012. *Asia Pac J Clin Oncol* (2013) 9(3):199-213. doi: 10.1111/ajco.12127.
3. Australian Institute of Health and Welfare. Cancer in Australia 2014: Actual incidence data from 1982 to 2011 and mortality data from 1982 to 2012 with projections to 2014. *Asia Pac J Clin Oncol* (2015) 11(3):208-20. doi: 10.1111/ajco.12407.
4. Australian Institute of Health and Welfare. Cancer in Australia: Actual incidence data from 1982 to 2013 and mortality data from 1982 to 2014 with projections to 2017. *Asia Pac J Clin Oncol* (2018) 14(1):5-15. doi: 10.1111/ajco.12761.
5. Baade PD, Turrell G, Aitken JF. A multilevel study of the determinants of area-level inequalities in colorectal cancer survival. *BMC Cancer* (2010) 10. doi: 10.1186/1471-2407-10-24.
6. Banham D, Roder D, Brown A. Comorbidities contribute to the risk of cancer death among Aboriginal and non-Aboriginal South Australians: Analysis of a matched cohort study. *Cancer Epidemiol* (2018) 52:75-82. doi: 10.1016/j.canep.2017.12.005.
7. Banham D, Roder D, Eckert M, Howard NJ, Canuto K, Brown A, et al. Cancer treatment and the risk of cancer death among Aboriginal and non-Aboriginal South Australians: analysis of a matched cohort study. *BMC Health Serv Res* (2019) 19(1):771. doi: 10.1186/s12913-019-4534-y.
8. Barraclough KA, Grace BS, Lawton P, McDonald SP. Residential Location and Kidney Transplant Outcomes in Indigenous Compared With Nonindigenous Australians. *Transplantation* (2016) 100(10):2168-76. doi: 10.1097/TP.0000000000001007.
9. Clark PJ, Stuart KA, Leggett BA, Crawford DH, Boyd P, Fawcett J, et al. Remoteness, race and social disadvantage: disparities in hepatocellular carcinoma incidence and survival in Queensland, Australia. *Liv Int* (2015) 35(12):2584-94. doi: 10.1111/liv.12853.
10. Condon JR, Garvey G, Whop LJ, Valery PC, Thomas D, Gruen R, et al. Aboriginal and Torres Strait Islander Australians and Cancer. *Cancer Forum* (2013) 37(1):27-30.
11. Davidson PM, Jiwa M, DiGiacomo ML, McGrath SJ, Newton PJ, Durey AJ, et al. The experience of lung cancer in Aboriginal and Torres Strait Islander peoples and what it means for policy, service planning and delivery. *Aust Health Rev* (2013) 37(1):70-8. doi: 10.1071/AH10955.
12. Davies A, Gurney J, Garvey G, Diaz A, Segelov E. Cancer care disparities among Australian and Aotearoa New Zealand Indigenous peoples. *Curr Opin Support Palliat Care* (2021) 15(3):162-8. doi: 10.1097/SPC.0000000000000558.
13. Diaz A, Whop LJ, Valery PC, Moore SP, Cunningham J, Garvey G, et al. Cancer outcomes for Aboriginal and Torres Strait Islander Australians in rural and remote areas. *Aust J Rural Health* (2015) 23(1):4-18. doi: 10.1111/ajr.12169.
14. Fitzadam S, Lin EM, Creighton N, Currow DC. Lung, breast and bowel cancer treatment for Aboriginal people in New South Wales: a population-based cohort study. *Intern Med J* (2021) 51(6):879-90. doi: 10.1111/imj.14967.
15. Gibberd A, Supramaniam R, Dillon A, Armstrong BK, O'Connell DL. Are Aboriginal people more likely to be diagnosed with more advanced cancer? *Med J Aust* (2015) 202(4):195-9. doi: 10.5694/mja14.00701.
16. Haggar FA, Pereira G, Preen DD, Holman CDJ, Einarsdottir K. Cancer Survival and Excess Mortality Estimates among Adolescents and Young Adults in Western Australia, 1982-2004: A Population-Based Study. *PLoS One* (2013) 8(2). doi: 10.1371/journal.pone.0055630.
17. Ho V, Whiteman D, Miller M, Raulli A, Ombiga J, Boyd P. Esophageal cancer in Indigenous Australians in Far North Queensland. *J Gastroenterol Hepatol* (2009) 24(10):1683-6. Epub 2009/10/03. doi: 10.1111/j.1440-1746.2009.05897.x.
18. Hopkins R, Dolan KA. Trends in cancer incidence and survival for indigenous and non-indigenous people in the Northern Territory. *Med J Aust* (2017) 207(1):46-.e1. doi: 10.5694/mja16.01390.
19. Khan A, Dale T, Martin H, Spalding L, Redfern C, Redfern A. The impact of site of metastasis on overall survival in indigenous and non-indigenous patients of Western Australia with breast cancer. *Ann Oncol* (2020) 31:S1265-S. doi: 10.1016/j.annonc.2020.10.079.

20. Khan A, Martin H, Spalding L, Redfern A. Survival outcome of indigenous and non-indigenous women of Western Australia with breast cancer in relation to remoteness. *Ann Oncol* (2020) 31:S71-S. doi: 10.1016/j.annonc.2020.03.254.
21. Khan A, Martin H, Spalding L, Redfern A. The impact of baseline modified glasgow prognostic score (mGPS) on survival outcomes in in indigenous and nonindigenous patients with advanced breast cancer patients of Western Australia. *Cancer Res* (2021) 81(4).
22. Luke C, Price T, Roder D. Epidemiology of cancer of the liver and intrahepatic bile ducts in an Australian population. *Asian Pac J Cancer Prev* (2010) 11(6):1479-85.
23. Martin JH, Coory MD, Valery PC, Green AC. Association of diabetes with survival among cohorts of Indigenous and non-Indigenous Australians with cancer. *Cancer Causes Control* (2009) 20(3):355-60. doi: 10.1007/s10552-008-9249-z.
24. Parker C, Tong SYC, Dempsey K, Condon J, Sharma SK, Chen JWC, et al. Hepatocellular carcinoma in Australia's Northern Territory: high incidence and poor outcome. *Med J Aust* (2014) 201(8):470-4. doi: 10.5694/mja13.11117.
25. Read DJ, Hayes I. Do Indigenous patients in Australia's Northern Territory present with more advanced colorectal cancer? A cohort study based on registry data. *ANZ J Surg* (2019) 89(10):1296-301. doi: 10.1111/ans.15376.
26. Read DJ, Frentzas S, Ward L, De Ieso P, Chen S, Devi V. Do histopathological features of breast cancer in Australian Indigenous women explain the survival disparity? A two decade long study in the Northern Territory. *Asia Pac J Clin Oncol* (2020) 16(6):348-55. doi: 10.1111/ajco.13377.
27. Roder D, Zorbas HM, Kollias J, Pyke CM, Walters D, Campbell ID, et al. Analysing risk factors for poorer breast cancer outcomes in residents of lower socioeconomic areas of Australia. *Aust Health Rev* (2014) 38(2):134-41. doi: 10.1071/ah13080.
28. Tan JYA, Otty ZA, Vangaveti VN, Buttner P, Varma SC, Joshi AJ, et al. A prospective comparison of times to presentation and treatment of regional and remote head and neck patients in North Queensland, Australia. *Intern Med J* (2016) 46(8):917-24. doi: 10.1111/imj.13138.
29. Tapia KA, Garvey G, Mc Entee M, Rickard M, Brennan P. Breast Cancer in Australian Indigenous Women: Incidence, Mortality, and Risk Factors. *Asian Pac J Cancer Prev* (2017) 18(4):873-84.
30. Tervonen HE, Purdie S, Creighton N. Using data linkage to enhance the reporting of cancer outcomes of Aboriginal and Torres Strait Islander people in NSW, Australia. *BMC Med Res Methodol* (2019) 19(1):245. doi: 10.1186/s12874-019-0884-8.
31. Tomita Y, Karapetis CS, Roder D, Beeke C, Hocking C, Roy AC, et al. Comparable survival outcome of metastatic colorectal cancer in Indigenous and non-Indigenous patients: Retrospective analysis of the South Australian metastatic colorectal cancer registry. *Aust J Rural Health* (2016) 24(2):85-91. doi: 10.1111/ajr.12219.
32. Withrow DR, Racey CS, Jamal S. A critical review of methods for assessing cancer survival disparities in indigenous population. *Ann Epidemiol* (2016) 26(8):579-91. doi: 10.1016/j.annepidem.2016.06.007.
33. Baade PD, Dasgupta P, Dickman PW, Cramb S, Williamson JD, Condon JR, et al. Quantifying the changes in survival inequality for Indigenous people diagnosed with cancer in Queensland, Australia. *Cancer Epidemiol* (2016) 43:1-8. doi: 10.1016/j.canep.2016.05.002.
34. Banham D, Roder D, Keefe D, Farshid G, Eckert M, Cargo M, et al. Disparities in cancer stage at diagnosis and survival of Aboriginal and non-Aboriginal South Australians. *Cancer Epidemiol* (2017) 48:131-9. Epub 2017/05/17. doi: 10.1016/j.canep.2017.04.013.
35. Banham D, Roder D, Keefe D, Farshid G, Eckert M, Howard N, et al. Disparities in breast screening, stage at diagnosis, cancer treatment and the subsequent risk of cancer death: a retrospective, matched cohort of aboriginal and non-aboriginal women with breast cancer. *BMC Health Serv Res* (2019) 19(1):387. doi: 10.1186/s12913-019-4147-5.
36. Basnayake TL, Valery PC, Carson P, De Ieso PB. Treatment and outcomes for indigenous and non-indigenous lung cancer patients in the Top End of the Northern Territory. *Intern Med J* (2021) 51(7):1081-91. doi: 10.1111/imj.14961.
37. Chong A, Roder D. Exploring differences in survival from cancer among Indigenous and non-Indigenous Australians: implications for health service delivery and research. *Asian Pac J Cancer Prev* (2010) 11(4):953-61.
38. Condon JR, Zhang X, Baade P, Griffiths K, Cunningham J, Roder DM, et al. Cancer survival for Aboriginal and Torres Strait Islander Australians: A national study of survival rates and excess mortality. *Popul Heath Metr* (2014) 12(1). doi: 10.1186/1478-7954-12-1.
39. Condon JR, Zhang X, Dempsey K, Garling L, Guthridge S. Trends in cancer incidence and survival for Indigenous and non-Indigenous people in the Northern Territory. *Med J Aust* (2016) 205(10):454-8. doi: 10.5694/mja16.00588.

40. Coory MD, Green AC, Stirling J, Valery PC. Survival of Indigenous and non-Indigenous Queenslanders after a diagnosis of lung cancer: a matched cohort study. *Med J Aust* (2008) 188(10):562-6. doi: 10.5694/j.1326-5377.2008.tb01790.x.
41. Cramb SM, Garvey G, Valery PC, Williamson JD, Baade PD, Cramb SM, et al. The first year counts: cancer survival among Indigenous and non-Indigenous Queenslanders, 1997-2006. *Med J Aust* (2012) 196(4):270-4. doi: 10.5694/mja11.11194.
42. Dasgupta P, Baade PD, Aitken JF, Turrell G. Multilevel determinants of breast cancer survival: association with geographic remoteness and area-level socioeconomic disadvantage. *Breast Cancer Res Treat* (2012) 132(2):701-10. doi: 10.1007/s10549-011-1899-y.
43. Diaz A, Moore SP, Martin JH, Green AC, Garvey G, Valery PC. Factors associated with cancer-specific and overall survival among Indigenous and non-Indigenous gynecologic cancer patients in Queensland, Australia: a matched cohort study. *Int J Gynecol Cancer* (2015) 25(3):542-7. Epub 2015/02/24. doi: 10.1097/igc.0000000000000375.
44. Diaz A, Baade PD, Valery PC, Whop LJ, Moore SP, Cunningham J, et al. Comorbidity and cervical cancer survival of Indigenous and non-Indigenous Australian women: A semi-national registry-based cohort study (2003-2012). *PLoS One* (2018) 13(5):e0196764. Epub 2018/05/09. doi: 10.1371/journal.pone.0196764.
45. Gibberd A, Supramaniam R, Dillon A, Armstrong BK, O'Connell DL. Lung cancer treatment and mortality for Aboriginal people in New South Wales, Australia: results from a population-based record linkage study and medical record audit. *BMC Cancer* (2016) 16. doi: 10.1186/s12885-016-2322-1.
46. He VYF, Condon JR, Baade PD, Zhang X, Zhao Y. Different survival analysis methods for measuring long-term outcomes of Indigenous and non-Indigenous Australian cancer patients in the presence and absence of competing risks. *Popul Heath Metr* (2017) 15(1). doi: 10.1186/s12963-016-0118-9.
47. Hsieh JCF, Cramb SM, McGree JM, Dunn NAM, Baade PD, Mengersen KL. Does geographic location impact the survival differential between screen- and interval-detected breast cancers? *Stoch Environ Res Risk Assess* (2016) 30(1):155-65. doi: 10.1007/s00477-015-1050-4.
48. Luke C, Tracey E, Stapleton A, Roder D. Exploring contrary trends in bladder cancer incidence, mortality and survival: implications for research and cancer control. *Intern Med J* (2010) 40(5):357-62. doi: 10.1111/j.1445-5994.2009.01980.x.
49. Moore SP, Green AC, Garvey G, Coory MD, Valery PC. A study of head and neck cancer treatment and survival among indigenous and non-indigenous people in Queensland, Australia, 1998 to 2004. *BMC Cancer* (2011) 11. doi: 10.1186/1471-2407-11-460.
50. Moore SP, Soerjomataram I, Green AC, Garvey G, Martin J, Valery PC. Breast cancer diagnosis, patterns of care and burden of disease in Queensland, Australia (1998-2004): does being Indigenous make a difference? *Int J Public Heath* (2016) 61(4):435-42. doi: 10.1007/s00038-015-0739-y.
51. Moore SP, Green AC, Bray F, Coory M, Garvey G, Sabesan S, et al. Colorectal cancer among Indigenous and non-Indigenous people in Queensland, Australia: Toward survival equality. *Asia Pac J Clin Oncol* (2016) 12(2):E209-E14. doi: 10.1111/ajco.12164.
52. Morrell S, You H, Baker D. Estimates of cancer incidence, mortality and survival in aboriginal people from NSW, Australia. *BMC Cancer* (2012) 12(1). doi: 10.1186/1471-2407-12-168.
53. Peng Y, Baade P. Survival disparities among recently diagnosed Aboriginal and Torres Strait Islander cancer patients in Australia remain. *Cancer Causes Control* (2021) 32(11):1315-20. doi: 10.1007/s10552-021-01474-6.
54. Pule L, Buckley E, Niyonsenga T, Banham D, Roder D. Developing a comorbidity index for comparing cancer outcomes in Aboriginal and non-Aboriginal Australians. *BMC Health Serv Res* (2018) 18. doi: 10.1186/s12913-018-3603-y.
55. Roder D, Webster F, Zorbas H, Sinclair S. Breast Screening and Breast Cancer Survival in Aboriginal and Torres Strait Islander Women of Australia. *Asian Pac J Cancer Prev* (2012) 13(1):147-55. doi: 10.7314/APJCP.2012.13.1.147.
56. Rodger JC, Supramaniam R, Gibberd AJ, Smith DP, Armstrong BK, Dillon A, et al. Prostate cancer mortality outcomes and patterns of primary treatment for Aboriginal men in New South Wales, Australia. *BJU Int* (2015) 115:16-23. doi: 10.1111/bju.12899.
57. Supramaniam R, Gibberd A, Dillon A, Goldsbury DE, O'Connell DL. Increasing rates of surgical treatment and preventing comorbidities may increase breast cancer survival for Aboriginal women. *BMC Cancer* (2014) 14:163. Epub 2014/03/13. doi: 10.1186/1471-2407-14-163.
58. Tervonen HE, Aranda S, Roder D, Walton R, Baker D, You H, et al. Differences in impact of Aboriginal and Torres Strait Islander status on cancer stage and survival by level of socio-economic disadvantage and remoteness of residence—A population-based cohort study in Australia. *Cancer Epidemiol* (2016) 41:132-8. doi: 10.1016/j.canep.2016.02.006.

59. Tervonen HE, Walton R, You H, Baker D, Roder D, Currow D, et al. After accounting for competing causes of death and more advanced stage, do Aboriginal and Torres Strait Islander peoples with cancer still have worse survival? A population-based cohort study in New South Wales. *BMC Cancer* (2017) 17(1):398. doi: 10.1186/s12885-017-3374-6.
60. Weir K, Supramaniam R, Gibberd A, Dillon A, Armstrong BK, O'Connell DL. Comparing colorectal cancer treatment and survival for Aboriginal and non-Aboriginal people in New South Wales. *Med J Aust* (2016) 204(4):56.e1-.e8. doi: 10.5694/mja15.01153.
61. Wigg AJ, Narayana SK, Hartel G, Medlin L, Pratt G, Powell EE, et al. Hepatocellular carcinoma amongst Aboriginal and Torres Strait Islander peoples of Australia. *EClinicalMedicine* (2021) 36:100919. doi: 10.1016/j.eclinm.2021.100919.
62. Youlden DR, Baade PD, Walker R, Pyke CM, Roder DM, Aitken JF. Breast Cancer Incidence and Survival Among Young Females in Queensland, Australia. *J Adolesc Young Adult Oncol* (2020) 9(3):402-9. doi: 10.1089/jayao.2019.0119.
